# Moderators, mediators, and components of a standalone smartphone application for postpartum depression: secondary analysis of a randomized controlled trial

**DOI:** 10.1007/s00737-026-01705-2

**Published:** 2026-05-22

**Authors:** Tatiane Borja, Pedro Fonseca Zuccolo, Leonardo Seda, Alicia Matijasevich, Guilherme Vanoni Polanczyk, Daniel Fatori

**Affiliations:** 1 Departamento de Psiquiatria, Faculdade de Medicina FMUSP, Universidade de São Paulo, São Paulo, Brazil; 2 Laboratório de Psicopatologia e Terapêutica Psiquiátrica LIM-23, Instituto de Psiquiatria, Hospital das Clinicas HCFMUSP, Faculdade de Medicina, Universidade de São Paulo, São Paulo, Brazil; 3 Departamento de Medicina Preventiva , Faculdade de Medicina FMUSP, Universidade de São Paulo, São Paulo, Brazil

## Abstract

**Purpose:**

The purpose of this study was to identify potential moderators, mediators, and efficacious components of a standalone app intervention on postpartum depression symptoms and functional impairment.

**Methods:**

A total of 264 women were randomized to intervention (Motherly app) or to an active control group. We analyzed depressive symptoms assessed by the Edinburgh Postnatal Depression Scale and functional impairment by the Clinical Global Impression at post-treatment as outcomes. Various potential moderators were assessed at baseline. Potential mediation variables were the level of behavioral activation (BA) measured by the Behavioral Activation for Depression Scale - Short Form and the Response-contingent positive reinforcement (RCPR) by the Reward Probability Index, at post-treatment. Intervention component analysis was evaluated using app engagement data.

**Results:**

The results indicate that intervention effects on depressive symptoms were moderated by the number of children and pediatric consultations, with stronger effects observed among mothers with fewer children and higher consultation frequency. Functional impairment was moderated by time spent with the child, with greater improvements among mothers with lower caregiving demands. Mediation analyses showed BA and RCPR mediated the effect of our intervention on depressive symptoms and functional impairment. Total and BA achievements were significantly associated with reduction in depressive symptoms.

**Conclusion:**

Our findings suggest that our app intervention efficacy is influenced by maternal characteristics and childcare responsibilities. BA and RCPR are key mechanisms, and higher engagement with app components produce greater symptom reduction.

**Supplementary Information:**

The online version contains supplementary material available at 10.1007/s00737-026-01705-2.

## Article highlights

We used data from a randomized controlled trial to identify moderators, mediators, and components of an app intervention for postpartum depression;

Maternal characteristics and childcare responsibilities moderated the effect of the intervention;

Levels of behavioral activation and response-contingent positive reinforcement mediated the effect of the intervention;

Higher engagement with the app, especially BA components, produced greater symptom reduction.

**Supplementary Information:**

The online version contains supplementary material available at 10.1007/s00737-026-01705-2.

## Introduction

Postpartum depression (PPD) is the most prevalent mental disorder in the postnatal period (Gelaye et al. 2016; Wang et al. 2021). Pharmacological treatment usually involves medications approved for Major Depressive Disorder, such as Selective serotonin reuptake inhibitors (Marx et al. 2023), although current evidence regarding their effectiveness and safety for managing PPD symptoms remains limited, particularly among women with more severe depression (Brown et al. 2021; De Crescenzo et al. 2014). Specific medications aligned with PPD pathophysiology (e.g., Zuranolone and Brexanolone) have demonstrated strong evidence of efficacy, but remain unregulated in most countries (Deligiannidis et al. 2021; Powell et al. 2020). Consequently, non-pharmacological interventions have emerged as viable alternatives (Nillni et al. 2018), with smartphone applications (app) gaining prominence for their acceptability and utility in symptom management (Hussain-Shamsy et al. 2020; Zhou et al. 2020), offering advantages including overcoming stigma, anonymity, flexibility, and accessibility for women (Andersson and Titov 2014; Lal and Adair 2014).

Although studies report efficacy of apps for PPD (Hussain-Shamsy et al. 2020; Zhou et al. 2020), a recent meta-analysis suggests a lack of a significant effect on depression symptoms and low quality of evidence (Tsai et al. 2022). Given this scenario and the early stage of this field, it is crucial to identify for whom and through which mechanism these interventions work by identifying moderators and mediators (Domhardt et al. 2021; Hayes 2022; Kraemer et al. 2002; Preacher, K. J., & Hayes, A. F., 2008), as well as the most efficacious components of the intervention. This knowledge can guide the optimization of apps (Huckvale et al. 2020) by prioritizing evidence-based features and refining existing programs.

However, current literature offers a scarcity of studies evaluating moderators, mediators, and components of app interventions for PPD (Domhardt et al. 2021; Feldman et al. 2021; Zhang et al. 2017). Addressing this gap, our study aims to identify the potential moderators and mediators of the effects of a standalone smartphone app on PPD symptoms and functional impairment in the context of a large randomized controlled trial (RCT). In addition, we sought to identify the most efficacious components of our intervention.

## Methods

### Study design and participants

This study is a secondary analysis of an RCT (NCT05055674) that investigated the efficacy of an app named Motherly in reducing depressive symptoms (Zuccolo et al. 2024). The results showed that the intervention did not significantly reduce depressive symptoms compared to the active control, but did improve sleep quality, behavioral activation, parental competence, and clinical status at post-treatment. Additional details of our study can be found elsewhere (Zuccolo et al. 2024).

### Procedures

A total of 264 participants were randomized into two groups: Motherly app (intervention group) and the COMVC app (active control group), using a computer-generated sequence implemented in REDCap, ensuring concealed and automatic allocation after baseline. Participants were informed that the study evaluated two apps without disclosing their names. Assessments were conducted at baseline (T1), post-treatment (T2, four weeks later), and follow-up (T3, four weeks post-treatment) by blinded interviewers (trained psychologists), with reassignment if blinding was compromised.

### Intervention

The Motherly app delivers psychoeducational information related to mental health, childcare, and healthy habits, combined with tools for scheduling daily activities that help reduce depressive symptoms, checklists, and notifications. The app applies gamification techniques (points, badges, and progress platforms) to promote engagement and healthy behavior change (Wu et al. 2021). Mental health modules included Behavioral Activation (BA), emotional regulation, sleep hygiene, cognitive restructuring and stress management (Zuccolo et al. 2024). The BA module was the primary intervention, based on the premise that a depressive environment is characterized by excessive aversive stimuli (punishers and negative reinforcers) and reduced positive reinforcement stimuli, creating a cyclical pattern (Lejuez et al. 2001). The intervention aims to enhance engagement in value-based activities to increase response-contingent positive reinforcement (RCPR) and reduce avoidance behavior, ultimately improving mood over time(Abreu and Abreu 2020; Kanter et al. 2010; Lejuez et al. 2001; Manos et al. 2010). Core components of BA include identifying personal values, planning contextualized activities, training reinforcement skills, relaxation, contingency management, and reducing avoidance behaviors (Kanter et al. 2010; Manos et al. 2010).

### Active control

In contrast to Motherly app, which provides tailored content specifically targeting PPD and actively engages mothers in healthy behaviors, the COMVC app provided to the active control group, delivers psychoeducational videos on general mental health, and conducts weekly symptom monitoring. Participants received a behavior timeline graph and corresponding videos based on symptom intensity (absent/mild, moderate/severe).

### Outcomes

PPD symptoms and functional impairment were the primary outcomes. PPD symptoms were measured by the EPDS, a well known 10-item scale (Cox et al. 1987; Santos et al. 2007). Functional impairment at T2 was evaluated using the Clinical Global Impression-Improvement scale (CGI-I), which rates clinical change from baseline on a seven-point scale (Busner and Targum 2007; Lima et al. 2007).

### Moderators

The moderator is a variable present at baseline or preceding treatment that interacts with the intervention, influencing its effect on the outcome (Kraemer et al. 2002, 2008). In our study, potential moderators were individual characteristics collected at T1, categorized into six groups: maternal characteristics (age, maternal skin color, schooling, family income, number of previous children, relationship with the father), support network (father’s involvement in pregnancy, social support), smartphone usage habits (maternal care apps, health/mental health apps, post-COVID change in smartphone usage habits, app usage frequency, social media apps usage), child’s characteristics (child’s age, average time spent with the child), psychological style (ruminative thinking), and health care (number of prenatal consultations, number of consultations with the pediatrician) (Suppl.Table S1).

In the study, a high socioeconomic status and younger age were theorized to be associated with better performance in the intervention. The variable household income refers to the average monthly earnings of all residents in Brazilian reais (BRL). Relationships with the father were assessed based on parental relationship, classified as stable (continuous relationship or live together) or unstable (including cases where the father is unknown).

Social support was hypothesized that the high levels would be associated with advantages in the intervention. It was assessed using the Multidimensional Scale of Perceived Social Support (MSPSS), a three-item version which measures subjective social support from family, friends, and significant others (Sousa 2019; Zimet et al. 1988). Father’s involvement during pregnancy was analyzed using three response categories: not involved, slightly involved and highly involved.

Smartphone usage habits were assessed to hypothesize that participants with more frequent use of apps may benefit more from our intervention. Participants reported: (a) usage of maternal care apps (“do you use any specific smartphone app for mothers?” [yes or no]), (b) usage of health/mental health apps (“do you use any specific smartphone app for physical or mental health?” [yes or no]), (c) post-COVID change in smartphone usage habits (“compared to the period before social distancing or the COVID-19 pandemic, how do you evaluate your smartphone usage?“, response options: much less, less, about the same, more, much more), (d) apps usage frequency (participants were asked about the use of mainstream apps (e.g., YouTube, Netflix, Instagram), then they had to respond for each one: “I don’t use it; I use it rarely [once, twice, or three times a month]; I use it occasionally [at least once a week]; I use it frequently [most days of the week]; daily use”), and (e) social media apps usage (participants were asked about frequency of creating content and/or posting online on social media apps, with the same response categories.

We hypothesized that the child plays a role in hindering the promotion of the intervention, resulting in fewer benefits for the mother from the app. The variable average time spent with the child was measured based on the average hours the mother typically spent with the child over the past seven days.

The ruminative style was evaluated due to the conjecture that the low standard of response would be favored in the intervention. The Ruminative Response Scale measured the variable, a 10-item version (RRS-10) with likert-type items indicating more ruminative thinking (Torres 2008).

Medical care during the perinatal period was hypothesized to help the intervention’s efficacy. The number of prenatal consultations refers to the total number of appointments attended during the gestational period, while the number of consultations with the pediatrician corresponds to the number of pediatric visits the child had up to the interview.

Sociodemographic characteristics were examined as a priori moderators, as these variables are commonly investigated in moderation analyses (Kraemer et al. 2002). Given the limited evidence on moderators in app-based interventions for PPD, additional variables were explored as potential moderators (Sextl-Plötz et al. 2024).

### Mediators

The mediator is associated with the intervention and helps to explain the mechanism through which the intervention influences the outcome, potentially exerting a direct or interactive effect (Kraemer et al. 2002, 2008). BA has been consistently identified as a key mechanism of change in digital interventions for depression (Angerer et al. 2024; Domhardt et al. 2021). The potential mediators analyzed were levels of BA and avoidance behaviors, and RCPR at T2. The BA and avoidance behavior levels were assessed using the Behavioral Activation for Depression Scale - Short form (BADS-SF), which evaluates treatment effects over the past seven days. Higher scores (0–24) indicate greater activation, which is positively associated with the likelihood of reinforcement and quality of life, and negatively with depressive symptoms (Abreu and Abreu 2020; Manos et al. 2011). The RCPR was measured by the Reward Probability Index (RPI), which assesses the main components of depressive disorder treatment, including the number and availability of potential reinforcers, the ability to obtain reinforcement, and exposure to aversive events. Higher scores suggest an increased probability of reward and few environmental suppressors inhibiting access to reinforcement (Abreu and Abreu 2020; Carvalho et al. 2011). Total scores were used in the mediation analyses.

### Intervention components analysis

Motherly provided a gamified experience based on achievements (badges as rewards) that were unlocked upon reaching various milestones related to specific goals. Motherly component analysis was measured using achievement data automatically recorded from T1 to T2. Achievements were divided into five variables: (1) Total achievements; (2) Meditation; (3) BA; (4) Self-assessment; (5) Child health monitoring. For more information on Motherly’s achievements, see Suppl. Table S2.

### Statistical analyses

Moderation and mediation analyses were evaluated according to the Hayes model using the PROCESS macro statistical algorithm for R version 4.2 beta, which applies the Hayes model with a linear modeling approach and complete case analysis (Hayes 2022).

Simple moderation analyses were conducted with group (intervention and active control) as independent variables and the outcomes as dependent variables. Eighteen models were estimated. The effect of the moderator-independent variable interaction with the dependent variable is valid when *p* < 0.05 (Hayes 2022).

In the parallel mediation models, the intervention group was entered as the independent variable (X) and the outcomes as dependent variables (Y). Two models were tested. The direct effect (coefficient c’) represents the impact of X on Y, independent of the mediators. Meanwhile, the indirect effects (coefficient a_1_b_1_, coefficient a_2_b_2_) correspond to the portions of the effect mediated independently by M_1_ (Total score of BADS-SF) and M_2_ (Total scores of RPI*)*. The total effect is the sum of the direct and indirect effects (c = c’+a_1_b_1_+a_2_b_2_), reflecting the change if it included all the effects (Hayes 2022). Indirect effects were considered significant when the 95% bootstrapping confidence interval (5,000 samples) did not include zero (Hayes and Rockwood 2017).

In order to explore the treatment effects of Motherly, we used a linear model to examine the relationship between the Motherly component analysis variables and the outcomes. A p-value < 0.05 was considered statistically significant.

## Results

**Fig. 1:**
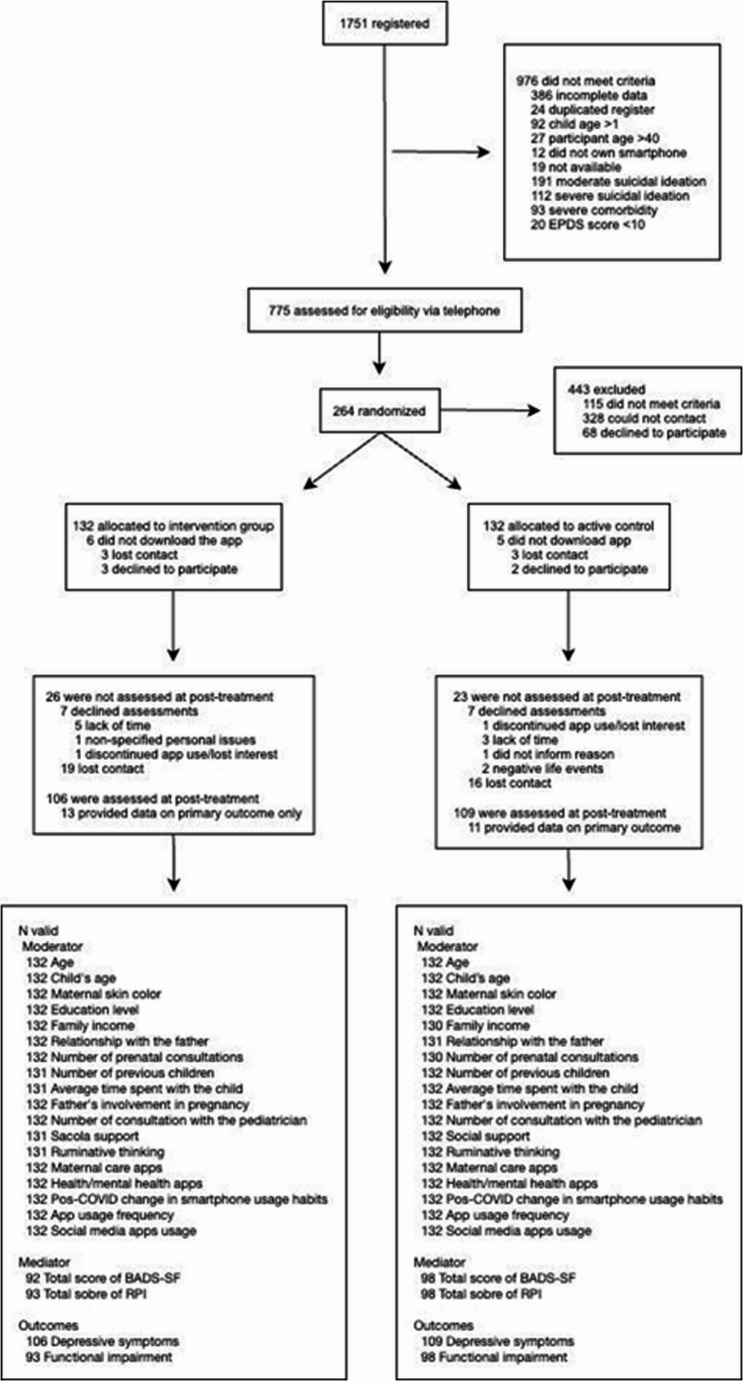
Participants’ flow in the study

Our study enrolled 1751 women with PPD and 264 met the study’s eligibility criteria (Fig. 1). Missingness patterns were examined (Supplementary Table S3) and were broadly comparable across the intervention and control groups, suggesting no evidence of substantial differential attrition.

Demographic and baseline characteristics were balanced between the groups (Table 1). The mean (SD) age was 31.16 (5.52) years; most of the patients were identified as white (66,29%) with a college degree or higher (61.74%). The majority (90.49%) were in a stable relationship with the father, and he was present in 71.97% of the pregnancies. The participants had an average of 11.28 prenatal consultations and 5.6 consultations with the pediatrician. The mean (SD) age of the baby was 161.62 days (101.61); the mothers spent about 12.26 h on average with the child and had 0.59 previous children. The average total score of EPDS was 17.31 (3.88).

**Table 1:** Baseline data for the Motherly Group (Intervention) and the COMVC Group (Active Control Group). Relative and absolute frequency (n, %), mean (M) and standard deviation (SD) of maternal sociodemographic data, information about the child, relationship with the father, health care and EPDS Total Score

Characteristics		Total	Group	
			Intervention (Motherly)	Active Control (COMVC)
Age, M (SD)		31.16 (5.52)	30.83 (5.67)	31.50 (5.35)
Maternal skin color, n (%)	White	175 (66.28%)	82 (62.12%)	93 (70.45%)
	Non-white	89 (33.71%)	50 (37.87%)	39 (29.54%)
Maternal educational level, n (%)	College degree or higher	163 (61.74%)	80 (60.60%)	83 (62.87%)
	others	101 (38.26%)	52 (39.39%)	49 (37.12%)
Family income (in thousands),(M(SD)		6,5 (6,8)	6,9 (6,9)	6,1 (6,6)
Relationship with the father, n (%)	Stable relationship	238 (90.49%)	119 (90.15%)	119 (90.83%)
	Unstable relationship	25 (9.51%)	13 (9.84%)	12 (9.16%)
Father’s involvement in pregnancy, n (%)	No involvement	17 (6.44%)	9 (6.81%)	8 (6.06%)
	Little involvement	57 (21.59%)	25 (18.93%)	32 (24.24%)
	Much involvement	190 (71.97%)	98 (74.24%)	92 (69.69%)
Number of prenatal consultations, M (SD)		11.28 (4.33)	10.96 (4.3)	11.6 (4.34)
Child’s age (in days), M (SD)		161.62 (101.61)	155.28 (93.04)	167.95 (109.50)
Number of previous children, M (SD)		0.59 (1.01)	0.46 (0.81)	0.71 (1.15)
Number of consultations with the pediatrician, M (SD)		5.60 (4.06)	5.27 (3.70)	5.93 (4.38)
Average time spent with the child (in hours), M (SD)		12.26 (5.25)	12.16 (4.90)	12.36 (5.58)
Psychological treatment, n (%)	No	136 (68%)	65 (69.89%)	71 (66.35%)
	Yes	64 (32%)	28 (30.10%)	36 (33.64%)
Psychiatric treatment, n (%)	No	69 (60%)	36 (69.23%)	33 (52.38%)
	Yes	46 (40%)	16 (30.76%)	30 (47.61%)
Total Score of EPDS, M (SD)		17.31 (3.88)	17.07(3.85)	17.56 (3.91)

### Moderators

Table 2 summarizes the simple moderation models. No statistically significant association was observed for moderators related to support network, psychological style, and smartphone usage habits. Among maternal characteristics, the number of previous children significantly moderated the intervention’s effect on depressive symptoms (B = 2.648, *p* < 0.001), with a progressive reduction in the intervention’s benefit as the number of previous children increased. In the child characteristics domain, average time spent with the child moderated the effect on functional impairment (B = −0.073, *p* = 0.033), such that mothers who spent less time with the child experienced greater improvements, whereas higher caregiving time was associated with smaller gains. In the healthcare domain, the number of pediatric consultations also moderated the effect of Motherly on depressive symptoms (B = −0.359, *p* = 0.038), indicating that the intervention effect was stronger among mothers with more frequent consultations, whereas this effect was attenuated among those with fewer consultations. Significant moderation effects are illustrated in Supplementary Figure S1.

**Table 2:** Simple moderation model using linear model showing association of depressive symptoms and functional impairment with moderating variables related to the Mother’s characteristics, Social support, Child’s characteristics, Psychological style, Healthcare, Smartphone usage habits

Moderator domain		Depressive symptoms			Functional impairment		
	Moderator	Beta	95% CI	*p*	Beta	95% CI	*p*
Maternal characteristics	Age	0.033	−0.208, 0.275	0.785	−0.044	−0.108, 0.019	0.169
	Maternal skin color	0.336	−2.484, 3.157	0.814	−0.025	−0.779, 0.728	0.946
	Maternal education level	0.274	−2.474, 3.024	0.844	−0.075	−0.813, 0.663	0.841
	Family income	0.000	−0.000, 0.000	0.552	−0.000	−0.000, 0.000	0.511
	Relationship with the father	1.047	−3.549, 5,644	0.653	−0.199	−1.383, 0.984	0.739
	Number of previous children	2.648	1.278, 4.018	< 0.001	−0.177	−0.624, 0.269	0.434
Support network	Father’s involvement in pregnancy	0.053	−2.330, 2.440	0.963	−0.111	−0.720, 0.497	0.717
	Social support	−0.151	−0.483, 0.179	0.367	0.019	−0.071, 0.109	0.672
Child’s characteristics	Child’s age	−0.002	−0.015, 0.011	0.723	−0.002	−0.005, 0.001	0.262
	Average time spent with the child	0.124	−0.128, 0.377	0.333	−0.073	−0.139, −0.006	0.032
Psychological style	Ruminative thinking	0.048	−0.231, 0.328	0.732	−0.011	−0.087, 0.064	0.764
Healthcare	Number of consultations with the pediatrician	−0.359	−0.700, −0.018	0.038	0.011	−0.081, 0.104	0.809
	Number of prenatal consultations	−0.019	−0.325, 0.286	0.900	0.064	−0.020, 0.148	0.134
Smartphone usage habits	Maternal care apps	−0.618	−3.285, 2.049	0.648	0.208	−0.501, 0.918	0.563
	Health/mental health apps	−1.830	−5.411, 1.751	0.314	−0.232	−1.187, 0.722	0.631
	Post-COVID change in smartphone usage habits	−0.259	−1.165, 0.646	0.572	−0.015	−0.254, 0.223	0.899
	App usage frequency	−0.123	−0.374, 0.128	0.335	0.008	−0.058, 0.076	0.797
	Social media apps usage	−0.454	−1.037, 0.128	0.126	−0.034	−0.193, 0.123	0.666

### Mediators

**Fig. 2:**
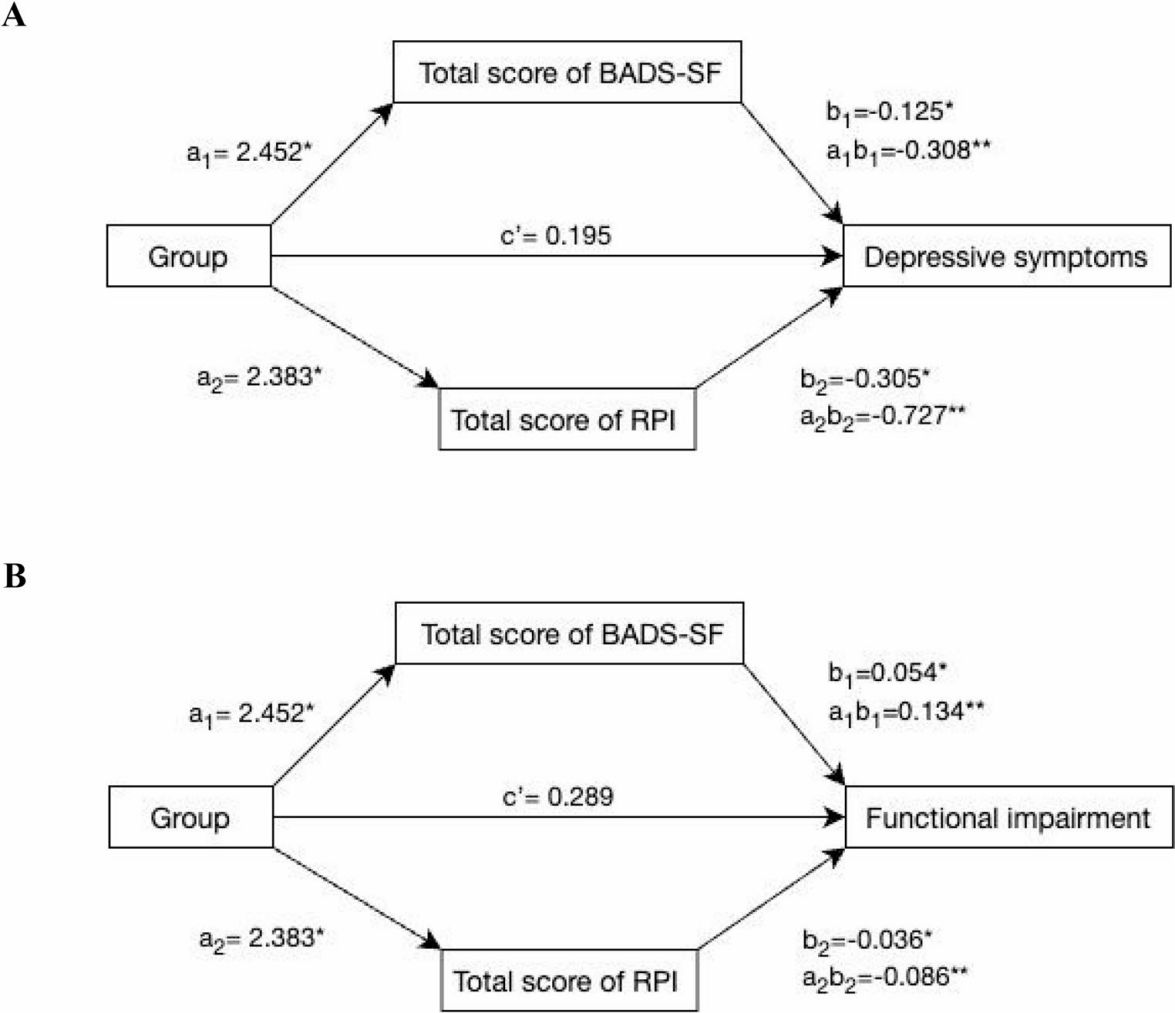
Diagram of the parallel mediation model of the BDAS-SF total score (M_1_) and RPI total score (M_2_) mediators in relation to treatment with Motherly and the outcomes, depressive symptoms (**A**) and functional impairment (**B**). ^a^a_1_b_1_= indirect effects of BADS-SF total score mediator; a_2_b_2_= indirect effect of the RPI total score mediator. *p-value< 0.05; **95% confidence interval valid without crossing zero

Figure 2 represents the parallel mediation model. Both tested mediators (BADS-SF, M_1_; RPI, M_2_) significantly mediated the association of Motherly with outcomes: depressive symptom (a_1_b_1_=−0.308, CI=−0.765, −0.004; a_2_b_2_=−0.727, CI=−1.447, −0.071) and functional impairment (a_1_b_1_=0.134, CI = 0.004, 0.280; a_2_b_2_=0.086, CI = 0.002, 0.205) (Suppl. Table S4).

### Intervention components

Suppl. Table S5 presents the frequency of achievements in the Motherly app, based on the 98 participants with available data and Table 3 presents the component analyses of the intervention. Total achievements (B=−0.381, *p* = 0.027) and BA achievements (B=−1.546, *p* = 0.030) were significantly associated with reduction in depressive symptoms. Other app achievements showed no significant association with either outcomes.

**Table 3:** Linear model showing association of depressive symptoms and functional impairment with Motherly component analysis variables (modules achievements)

Outcome	Intervention component analysis	Beta	SE	*p*
Depressive symptoms	Total achievements	−0.381	0.169	0.027
	Meditation	−0.166	0.714	0.816
	Behavioral activation	−1.546	0.702	0.030
	Self-assessment	−1.554	0.136	0.256
	Child health monitoring	−0.695	0.390	0.079
Functional impairment	Total achievements	0.077	0.046	0.097
	Meditation	0.094	0.191	0.622
	Behavioral activation	0.271	0.188	0.155
	Self-assessment	0.254	0.361	0.483
	Child health monitoring	0.121	0.107	0.264

## Discussion

This study used data from a large RCT to identify potential moderators, mediators, and key components contributing to the effects of an app (Motherly) on PPD symptoms. Our findings provide evidence of characteristics that may optimize outcomes and reveal mechanisms underlying the intervention’s effects. Our findings suggested that the number of pediatric consultations positively moderated the effects of our intervention, while the average time spent with the child and the number of children negatively moderated. Mediation analyses indicated that BA and RCPR levels, relevant psychological constructs linked to behavior changes, significantly mediated Motherly´s impact on outcomes. Additionally, we examined which elements were most influential in driving our intervention. Components analyses revealed that general app engagement and BA module engagement significantly influenced the effects of our intervention. To our knowledge, this is one of the few RCTs on maternal depression to report moderators, mediators, and components of an app intervention for PPD. Although the mean EPDS changes did not reach the threshold for reliable clinical change (≥ 4 points), these findings provide important insights into for whom and how the intervention works, which may help optimize and refine digital interventions for PPD in future implementations.

### Moderators

We found that a higher number of consultations with the pediatrician in the intervention group moderated the reduction of depressive symptoms. This suggests Motherly could be more appropriate when integrated into ongoing professional care. Pediatric visits in the first year postpartum are often the only consistent contact mothers have with health services, and they can provide support, screen for PPD, and facilitate referrals to mental health(Earls et al. 2019; Liberto 2012; Olson et al. 2006). However, a higher frequency of consultations may also reflect increased health concerns, comorbid conditions, or greater help-seeking behavior, and therefore this interpretation should be considered cautiously.

Our analysis confirmed our initial hypothesis: Motherly was more effective among mothers with fewer children and among those who spent less time with the child, moderating the improvement of depressive symptoms and functional impairment, respectively. Specifically, mothers with fewer children showed greater improvements in depressive symptoms following the intervention, whereas this benefit diminished as the number of children increased. Similarly, mothers who reported spending less time with the child showed greater improvements in functional impairment, whereas this effect was attenuated among those who spent more time in daily caregiving. These findings suggest that app-based interventions may be more effective when caregiving demands are lower. PPD is known to impair mother-infant bonding and reduce maternal responsiveness (Brummelte & Galea, 2016; Slomian et al., 2019). Additionally, having more children is related to increased parental stress and depressive symptoms due to the rising demands of caring for both previous children and newborns (Cho et al., 2022). Higher caregiving demands may limit the time and energy available to engage with digital therapeutic strategies. Notably, two of the three child-related variables influenced the intervention’s effect on PPD, suggesting that caregiving load and contextual constraints may play an important role in shaping treatment response.

We hypothesized that smartphone usage habits (e.g., use of maternal care apps, app usage frequency) could influence the engagement of our intervention. In this sense, the prior digital experience could be more prone to engaging with Motherly. However, it did not moderate our intervention effects in any outcomes. This may reflect the predominantly passive use of commercially available apps, which often lack scientific validation(John Touros 2017; Tucker et al. 2021) and may not prepare users for structured, evidence-based interventions like Motherly.

### Mediators

Motherly’s core strategy is BA, which aims to reduce depressive symptoms by increasing engagement in meaningful and positively reinforcing activities (Kanter et al. 2010). Using the BADS-SF and the RPI scales, we analyzed how Motherly, through BA techniques, could improve mood and the feeling of positive rewards from activities. We found that the level of BA and avoidance behaviors, and the RCPR mediated both outcomes. By promoting conditions that supported the activation, these mechanisms helped to reduce depressive symptoms and the severity of PPD. This finding is congruent with the BA model of depression and explains the mechanism by how our intervention achieved its effects. These findings reinforce the role of BA in app interventions, consistent with other studies (Alber et al. 2023; Huguet et al. 2016; Mancinelli et al. 2023).

### Intervention components analysis

Motherly provides achievements related to user engagement in specific activities (e.g., BA, child health monitoring). Total number of achievements was associated with reducing depression symptoms, suggesting a dose-response effect. In particular, achievements related to the BA module were also associated with symptom reduction, supporting the mechanism our intervention produced its effect, potentially guiding future app interventions for PPD. Mindfulness activity did not affect outcomes. Although standalone mindfulness apps can reduce depression (Gál et al. 2021; Min et al. 2023), in our study, efficacy may have been limited due to low engagement with the meditation component.

### Limitation

This study has some limitations. First, our moderation, mediation and components analyses were not pre-registered, although the outcomes evaluated were. Second, our original sample calculation was not based on such analyses. The effects of moderators, mediators and components on outcomes could be minor in terms of effect sizes, demanding larger sample sizes. However, our study presents a relatively large sample size, potentially mitigating the risk of type II errors. Third, the PROCESS macro excludes all cases with missing data, which may reduce statistical power and introduce bias if the data are not missing completely at random. Fourth, according to the Reliable Change Index (RCI) proposed by (Matthey 2004), a change of ≥ 4 points on the EPDS is typically required to indicate clinical significance, although all significant findings in our study were below the RCI threshold, suggesting a modest clinical impact of the intervention. Finally, since we did not measure other modules (e.g., cognitive restructuring, stress management), we could not assess the impact of these variables.

## Conclusion

We aimed to identify the moderators and mediators of Motherly’s effects on PPD, as well as its most efficacious components. Our results suggest that our app intervention may be more suited for women under professional healthcare, while higher childcare demands may hinder treatment efficacy. These results underscore the need to consider the caregiving context when implementing app interventions. Moreover, mediators of the effects of Motherly on outcomes were consistent with the BA model of depression, since levels of BA and RCPR mediated overall clinical improvement. Finally, app engagement was directly related to clinical improvement in depressive symptoms, while BA components contributed to reducing depressive symptoms, supporting the mechanism by which our app treats PPD. Overall, our findings provide relevant data on how to improve existing app interventions for PPD and develop new ones.

## Supplementary Information

Below is the link to the electronic supplementary material.

Supplementary Material 1 (DOCX 447 KB)

## Data Availability

No datasets were generated or analysed during the current study.
