# Supplementary material for: Moderators, mediators, and components of a standalone smartphone application for postpartum depression: secondary analysis of a randomized controlled trial
Source: Arch Womens Ment Health. 2026 May 22;29(3):85. doi: 10.1007/s00737-026-01705-2 (PMC13197248; doi:10.1007/s00737-026-01705-2)

**Supplementary Data**

| **Table S1.** Description of variables | | | |
| --- | --- | --- | --- |
|  | **Variables** |  | **Time-point** |
| **Moderator** | Maternal characteristics | Age | T1 |
|  |  | Maternal skin color | T1 |
|  |  | Maternal education level | T1 |
|  |  | Family income | T1 |
|  |  | Relationship with the father | T1 |
|  |  | Number of previous children | T1 |
|  | Support network | Father's involvement in pregnancy | T1 |
|  |  | Social support | T1 |
|  | Child's characteristics | Child's age | T1 |
|  |  | Average time spent with the child | T1 |
|  | Psychological style | Ruminative thinking | T1 |
|  | Healthcare | Number of consultations with the pediatrician | T1 |
|  |  | Number of prenatal consultations | T1 |
|  | Smartphone usage habits | Maternal care apps | T1 |
|  |  | Health/mental health apps | T1 |
|  |  | Post-COVID change in smartphone usage habits | T1 |
|  |  | App usage frequency | T1 |
|  |  | Social media apps usage | T1 |
| **Mediador** |  | Total score of BADS-SF | T2 |
|  |  | Total score of RPI | T2 |
| **Intervention Component** |  | Total achievements | T1 to T2 |
|  |  | Meditation | T1 to T2 |
|  |  | Behavioral activation | T1 to T2 |
|  |  | Self-assessment | T1 to T2 |
|  |  | Child health monitoring | T1 to T2 |

| **Table S2.** Criteria for accomplish the achievements in the Motherly app activities | | | | | | |
| --- | --- | --- | --- | --- | --- | --- |
| **Title of Achievement** | **Description** | **Meditation** | **Self-assessment** | **Child health monitoring** | **Behavioral**  **activation** | **Total achievements** |
| Mindfulness | Completed the first session of meditation | X |  |  |  | X |
| A Safe Place | Completed the meditation journey | X |  |  |  | X |
| Sensei Master | Completed 50 meditation audios | X |  |  |  | X |
| Behavioral Activation | Completed the first part of the tutorial (theoretical part of the mental health tutorial) |  |  |  |  | X |
| Behavioral Activation 2 | Completed the second part of the tutorial (practical part) |  |  |  |  | X |
| Basic Activation | Completed 5 behavioral activation activities |  |  |  | X | X |
| Intermediate Activation | Completed 20 behavioral activation activities |  |  |  | X | X |
| Advanced Activation | Completed 50 behavioral activation activities |  |  |  | X | X |
| Leisure | Completed 15 behavioral activation activities of the "Leisure" type |  |  |  |  | X |
| Productive | Completed 15 behavioral activation activities of the "Productivity" type |  |  |  |  | X |
| Related | Completed 15 behavioral activation activities of the "Relationships" type |  |  |  |  | X |
| Relaxation | Completed 1 relaxation session |  |  |  |  | X |
| Plenitude | Completed 10 relaxation sessions |  |  |  |  | X |
| Zen | Completed 50 relaxation sessions |  |  |  |  | X |
| Hygienic Sleep | Completed the sleep hygiene journey |  |  |  |  | X |
| My Mental Health | Filled out 1 mental health assessment |  | X |  |  | X |
| Mental Care | Filled out 10 mental health assessments |  | X |  |  | X |
| Disciplined Patient | Filled out 30 mental health assessments |  | X |  |  | X |
| Follow-up | Scheduled 1 medical appointment |  |  |  |  | X |
| Committed Patient | Scheduled 4 medical appointments |  |  |  |  | X |
| Aced Appointment | Scheduled 8 medical appointments |  |  |  |  | X |
| Medical Exams I | Scheduled 1 medical exam |  |  |  |  | X |
| Medical Exams II | Scheduled 3 medical exams |  |  |  |  | X |
| Medical Exams III | Scheduled 6 medical exams |  |  |  |  | X |
| Motherly | Completed the pregnancy |  |  |  |  | X |
| My First Milestone | Recorded the first developmental milestone of the child |  |  | X |  | X |
| Growing Up! | Recorded the 15 developmental milestone of the child |  |  | X |  | X |
| Little Gentleman/Lady | Recorded the 30 developmental milestone of the child |  |  | X |  | X |
| My First Vaccine | Scheduled 1 vaccine for a child |  |  | X |  | X |
| Vaccinated | Scheduled 8 vaccines for a child |  |  | X |  | X |
| Vaccines Up to Date! | Scheduled all vaccines for a child |  |  |  |  | X |
| Understanding Depression | Learned the theory about Behavioral Activation |  |  |  |  | X |
| Activating | Learned how to create an activity |  |  |  |  | X |
| Curious | Read 3 items of the library |  |  |  |  | X |
| Avid Reader | Read 25 items of the library |  |  |  |  | X |
| PhD in Pregnancy | Read 70 items of the library |  |  |  |  | X |

| **Table S3.** Frequency and percentage of missing data for moderator, mediator variables and outcome, according to time-point and group. | | | | | | | | | | |
| --- | --- | --- | --- | --- | --- | --- | --- | --- | --- | --- |
|  |  |  | **T1** | | | | **T2** | | | |
|  |  |  | **Motherly** | | **Active control** | | **Motherly** | | **Active control** | |
|  |  |  | **N** | **%** | **N** | **%** | **N** | **%** | **N** | **%** |
| **Moderator** | Age |  | 0 | 0 | 0 | 0 | - | - | - | - |
|  | Maternal skin color |  | 0 | 0 | 0 | 0 | - | - | - | - |
|  | Maternal education level |  | 0 | 0 | 0 | 0 | - | - | - | - |
|  | Family income |  | 0 | 0 | 2 | 1.51 | - | - | - | - |
|  | Relationship with the father |  | 0 | 0 | 1 | 0.75 | - | - | - | - |
|  | Number of previous children |  | 1 | 0.75 | 0 | 0 | - | - | - | - |
|  | Father's involvement in pregnancy |  | 0 | 0 | 0 | 0 | - | - | - | - |
|  | Social support |  | 1 | 0.75 | 0 | 0 | - | - | - | - |
|  | Child's age |  | 0 | 0 | 0 | 0 | - | - | - | - |
|  | Average time spent with the child |  | 1 | 0.75 | 0 | 0 | - | - | - | - |
|  | Ruminative thinking |  | 1 | 0.75 | 0 | 0 | - | - | - | - |
|  | Number of consultations with the pediatrician |  | 0 | 0 | 0 | 0 | - | - | - | - |
|  | Number of prenatal consultations |  | 0 | 0 | 2 | 1.51 | - | - | - | - |
|  | Maternal care apps |  | 0 | 0 | 0 | 0 | - | - | - | - |
|  | Health/mental health apps |  | 0 | 0 | 0 | 0 | - | - | - | - |
|  | Post-COVID change in smartphone usage habits |  | 0 | 0 | 0 | 0 | - | - | - | - |
|  | App usage frequency |  | 0 | 0 | 0 | 0 | - | - | - | - |
|  | Social media apps usage |  | 0 | 0 | 0 | 0 | - | - | - | - |
| **Mediator** | Total score of BADS-SF |  | - | - | - | - | 40 | 30.30 | 34 | 25.75 |
|  | Total score of RPI total |  | - | - | - | - | 39 | 29.54 | 34 | 25.75 |
| **Outcome** | Depression symptoms |  | 0 | 0 | 0 | 0 | 26 | 19.69 | 23 | 17.42 |
|  | Functional impairment |  | - | - | - | - | 39 | 29.54 | 34 | 25.75 |

| **Table S4.** Indirect, direct and total effects of the parallel mediation model testing the association between the Motherly app and potential parallel mediators with the outcomes, depressive symptoms and functional impairment. | | | | | | | | | | | | | | |
| --- | --- | --- | --- | --- | --- | --- | --- | --- | --- | --- | --- | --- | --- | --- |
| **Outcomes** | **Indirect Effect (a_1_b_1_)** | | **Effect a_1_** | | **Effect b_1_** | | **Effect c'** | | **Indirect Effect (a_2_b_2_)** | | **Effect a_2_** | | **Effect b_2_** | |
|  | **Beta** | **95% CI** | **Beta** | **95% CI** | **Beta** | **95% CI** | **Beta** | **95% CI** | **Beta** | **95% CI** | **Beta** | **95% CI** | **Beta** | **95% CI** |
| **Depressive symptoms** | -0.308 | -0.765, -0.004 | 2.452 | 0.120, 4.784 | -0.125 | -0.212, -0.038 | 0.195 | -0.877, 1.267 | -0.727 | -1.447, -0.071 | 2.383 | 0.239, 4.526 | -0.305 | -0.400, -0.210 |
| **Functional impairment** | 0.134 | 0.004, 0.280 | 2.452 | 0.120, 4.784 | 0.054 | 0.029, 0.079 | 0.289 | -0.017, 0.596 | 0.086 | 0.002, 0.205 | 2.383 | 0.239, 4.526 | 0.036 | 0.009, 0.063 |

^a^BADS total score=M_1_; RPI total score= M_2_; 95% CI=Bootstrap confidence interval of the mediation model; a_1_b_1_= indirect effects of BADS-SF total score mediator; a_2_b_2_= indirect effect of the RPI total score mediator

| **Table S5.** Achievement rates among participants in the Motherly app group (N=98). | | | | |
| --- | --- | --- | --- | --- |
|  | **Title of Achievement** | **Description** | **Frequency** | |
|  |  |  | **n** | **%** |
| **Meditation** | Mindfulness | Completed the first session of meditation | 28 | 28.57 |
|  | A Safe Place | Completed the meditation journey | 8 | 8.16 |
|  | Sensei Master | Completed 50 meditation audios | 3 | 3.06 |
| **Behavioral activation** | Basic Activation | Completed 5 behavioral activation activities | 16 | 16.33 |
|  | Intermediate Activation | Completed 20 behavioral activation activities | 6 | 6.12 |
|  | Advanced Activation | Completed 50 behavioral activation activities | 3 | 3.06 |
| **Self-assessment** | My Mental Health | Filled out 1 mental health assessment | 98 | 100 |
|  | Mental Care | Filled out 10 mental health assessments | 10 | 10.20 |
|  | Disciplined Patient | Filled out 30 mental health assessments | 1 | 1.02 |
| **Child health monitoring** | My First Milestone | Recorded the first developmental milestone of the child | 41 | 41.84 |
|  | Growing Up! | Recorded the 15 developmental milestone of the child | 37 | 37.76 |
|  | Little Gentleman/Lady | Recorded the 30 developmental milestone of the child | 3 | 3.06 |
|  | My First Vaccine | Scheduled 1 vaccine for a child | 12 | 12.24 |
|  | Vaccinated | Scheduled 8 vaccines for a child | 8 | 8.16 |
|  |  |  |  |  |

**Figure S1.** Moderation plots for the effects of the Motherly intervention. (A) Interaction between group and number of previous children on depressive symptoms; (B) Interaction between group and average time spent with the child on dysfunctional impairment; (C) Interaction between group and number of pediatric consultations on depressive symptoms.

A


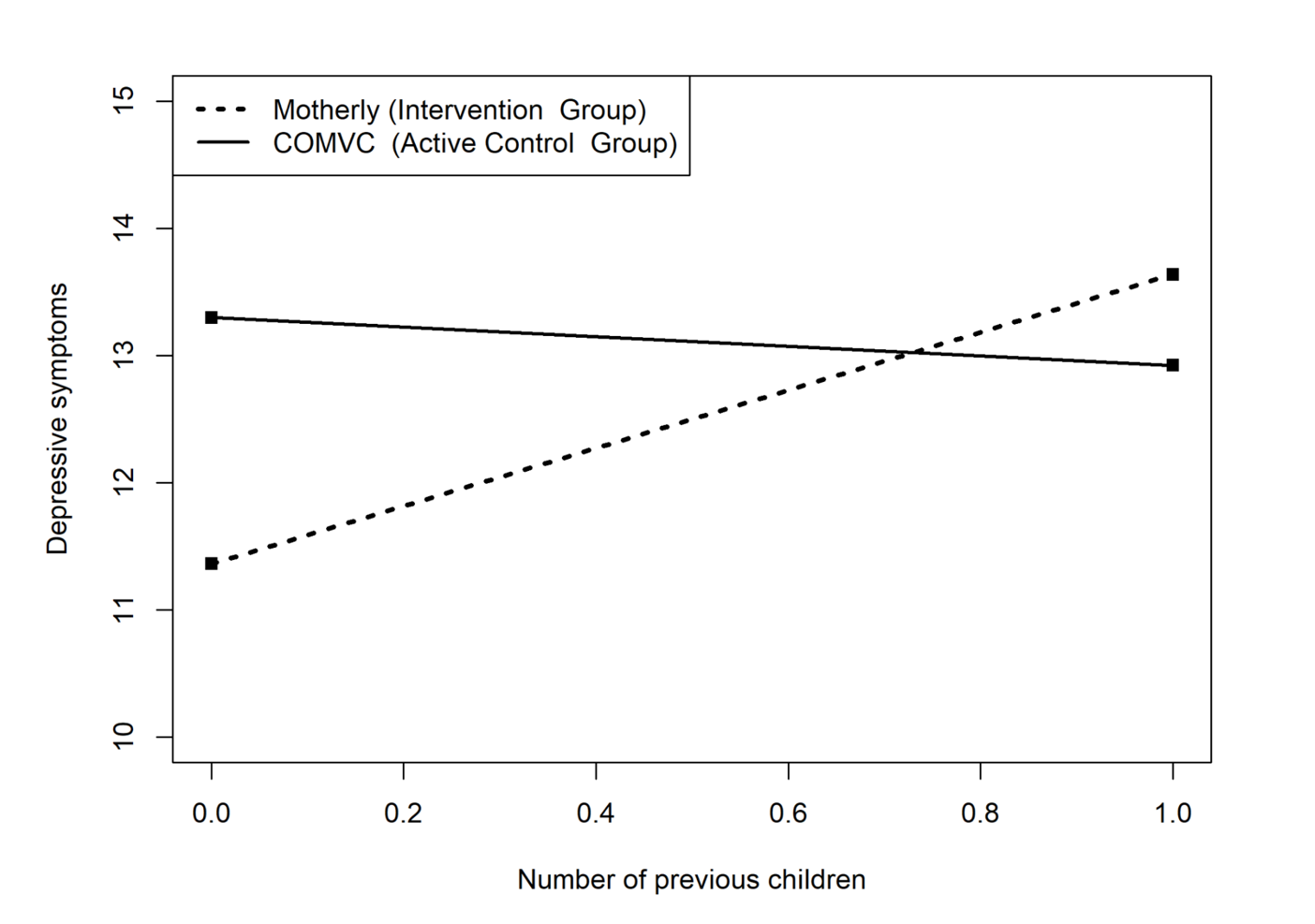


B


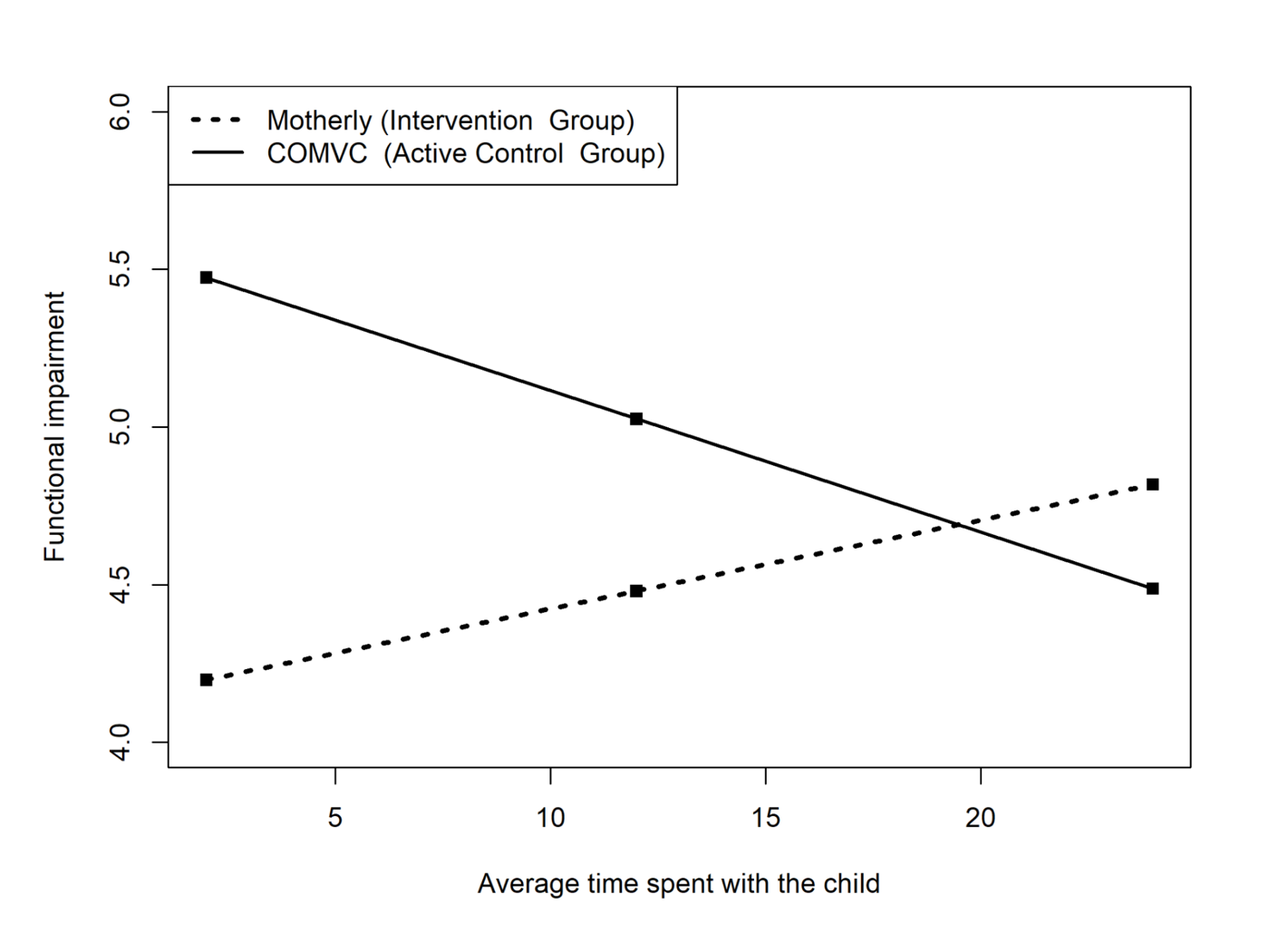


C


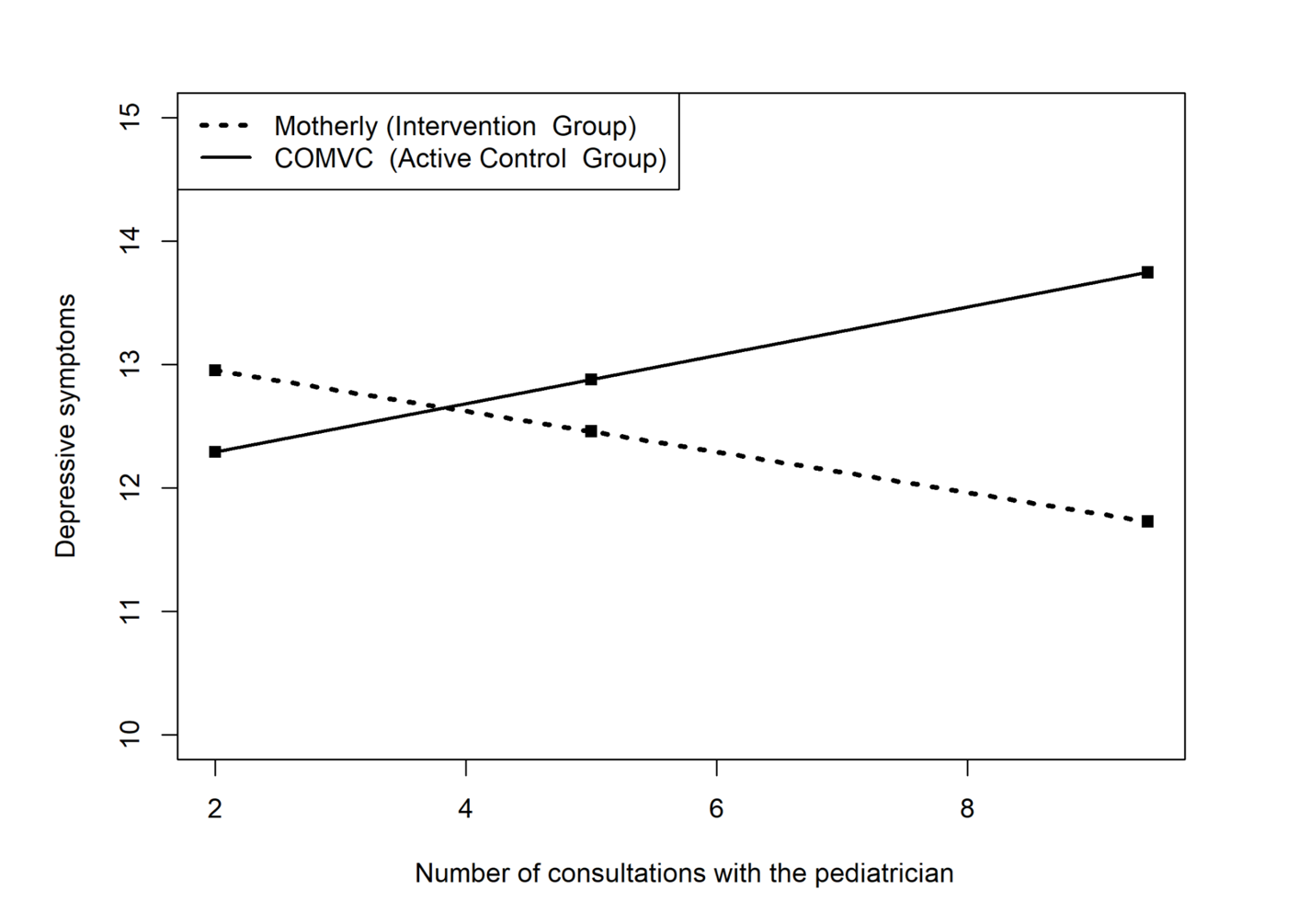

Supplement: Supplementary file 1 — Supplementary Material 1 (DOCX 447 KB) [file 737_2026_1705_MOESM1_ESM.docx]
